# Corneal Spherical Aberration and Corneal Asphericity after Small Incision Lenticule Extraction and Femtosecond Laser-Assisted LASIK

**DOI:** 10.1155/2017/4921090

**Published:** 2017-08-27

**Authors:** Hui Zhang, Yan Wang, Hua Li

**Affiliations:** ^1^Clinical College of Ophthalmology, Tianjin Medical University, Tianjin 300020, China; ^2^Tianjin Eye Hospital, Tianjin 300020, China; ^3^Tianjin Key Laboratory of Ophthalmology and Visual Science, Tianjin 300020, China; ^4^Tianjin Eye Institute, Tianjin 300020, China

## Abstract

**Purpose:**

To investigate corneal spherical aberration and corneal asphericity after small incision lenticule extraction (SMILE) and femtosecond laser-assisted laser in situ keratomileusis (FS-LASIK).

**Methods:**

This study enrolled 70 patients having SMILE and 64 subjects receiving FS-LASIK. The preoperative spherical equivalent (SE) was −5.83 ± 1.23 diopters (D) and −6.20 ± 1.52 D, respectively. The uncorrected distance visual acuity (UDVA), corrected distance visual acuity (CDVA), SE, corneal spherical aberration, and asphericity over the 6.0 mm cornea were evaluated preoperatively and postoperatively.

**Results:**

At 6 months, the UDVA, CDVA, and SE were −0.12 ± 0.11, −0.05 ± 0.05, and −0.16 ± 0.19 D in SMILE and −0.10 ± 0.06, −0.03 ± 0.06, and −0.08 ± 0.25 D in FS-LASIK. There was no difference between groups in the postoperative UDVA, CDVA, or SE (*P* > 0.05). SMILE showed lower inductions of spherical aberration along the anterior surface and the total cornea and less increases in corneal asphericity of the anterior surface postoperatively than FS-LASIK (*P* < 0.01). There were significant correlations between the changes in spherical aberration and corneal asphericity (*P* < 0.001).

**Conclusions:**

SMILE and FS-LASIK exhibited excellent visual results and refractive outcomes. SMILE induced less increase in corneal spherical aberration and better preserved the corneal asphericity of the anterior corneal surface than FS-LASIK. Corneal asphericity changes contributed to the corneal spherical aberration changes following SMILE and FS-LASIK.

## 1. Introduction

For the past few years, small incision lenticule extraction (SMILE) has become an increasingly popular corneal refractive surgery worldwide, with being approved by FDA of the United States recently. Although promising clinical results, in terms of safety, stability, predictability, and efficacy [1–3], were reported, an increase in the corneal spherical aberration was still found in patients undergoing SMILE procedure [4, 5]. As a result, the visual performance of the eye probably could be affected, because strong correlation between starburst and glare with spherical aberration was showed in previous study [6]. The increase in the corneal spherical aberration after SMILE was reported to be less than that after femtosecond laser-assisted laser in situ keratomileusis (FS-LASIK) [4, 5, 7]. However, the reason remained limited so far.

Corneal spherical aberration and corneal asphericity are proportionally related [8, 9]. The study was conducted to compare the change patterns of corneal spherical aberration and corneal asphericity between SMILE and FS-LASIK and investigate how much changes of corneal asphericity could account for the variation of corneal spherical aberration after SMILE and FS-LASIK procedures.

## 2. Methods

### 2.1. Subjects

The study was conducted in accordance with the tenets of the Declaration of Helsinki. Informed consent was obtained from each patient before refractive surgery and data collection. A total of 134 patients who underwent SMILE or FS-LASIK surgery at Tianjin Eye Hospital, China, participated in this study. The patients were asked about their myopia history and underwent an ophthalmologic examination including uncorrected distance visual acuity (UDVA), corrected distance visual acuity (CDVA), manifest and cycloplegic refractions, intraocular pressure (IOP), corneal topography, slit-lamp microscopy, and dilated indirect fundoscopy. Inclusion criteria were 18 years of age or older, stable refraction over the past year (≤0.50 diopter (D) progression), CDVA of 20/25 or better (0.1 logMAR or better), and IOP less than 21 mmHg. Exclusion criteria were estimated residual stromal thickness (RST) less than 250 *μ*m, keratoconus or suspected keratoconus, active ocular and systemic disease, and prior clinical history of ocular trauma or surgery. Soft contact lens wearers were advised to stop wearing their lenses at least two weeks before surgery.

### 2.2. Surgical Techniques

All surgeries were performed under topical anesthesia by the same surgeon to completely correct the preoperative manifest refractions.

SMILE is a fully flapless procedure during which only one type of laser is used. As described in detail by Sekundo et al. [10] and Shah et al. [11], the VisuMax femtosecond laser system (Carl Zeiss Meditec AG, Jena, Germany), with a repetition rate of 500 kHz, is used to create an intrastromal refractive lenticule and a small incision. The surgeon then separates the lenticule from the surrounding tissue and extracts it through the small incision. In this study, the diameter of the optical zone was 6.0 mm. A transition zone of 0.1 mm was induced in cases of astigmatism. The thickness and diameter of the cap were 110 *μ*m and 7.0 or 7.2 mm, respectively. A 3.0–5.0 mm incision was made through the superior cap margin. The femtosecond laser energy used during all SMILE procedures was 120–180 nJ.

In FS-LASIK, after a flap was created with the VisuMax femtosecond laser system (pulse energy of 120–130 nJ), the refractive ablation was performed with the Allegretto excimer laser system (WaveLight Laser Technologie AG, Erlangen, Germany). A 6.0 mm optical zone, surrounded by a transition zone of 1.0 mm, was applied to all eyes. The flap had a thickness of 100–110 *μ*m, a diameter of 8.0 mm, and a nasal hinge.

After surgery, topical ofloxacin 0.3% (Tarivid) was applied 4 time/day for three days. Fluorometholone 0.1% (Flumetholon, Santen) was administered 4 times daily with a taper over two weeks.

### 2.3. Measurements of Corneal Spherical Aberration and Corneal Asphericity

Pentacam HR topography with a 25-picture scan mode was used to determine corneal spherical aberration and corneal asphericity. Measurements were performed for patients' natural pupils under scotopic conditions. To minimize the potential effect of tear film on corneal imaging, patients were required to keep fixating on a target immediately after a blink. The Pentacam HR's camera then started rotating and scanning the cornea. Only measurements marked as “OK” in quality specification were considered to be valid.

Data for the study provided by the Pentacam HR topography were for a corneal diameter of 6.0 mm. Corneal spherical aberration was assessed for both the anterior and the posterior corneal surfaces, as well as the total cornea. Corneal asphericity was analyzed for both the anterior and the posterior corneal surfaces. The measurements were taken before and 1 and 6 months after SMILE and FS-LASIK.

### 2.4. Statistical Analysis

Data analysis was performed with IBM SPSS Statistics version 20.0 (IBM Corp., Armonk, NY). All data are represented as mean ± standard deviation (SD). Although most patients had bilateral surgical correction, only data from the right eyes were analyzed. The normality was tested with the Shapiro-Wilk test. One-way repeated measures analysis of variance (ANOVA) with post hoc Bonferroni testing was used to determine the changes over time in SMILE or FS-LASIK. Differences between SMILE and FS-LASIK were assessed with independent *t*-tests. In addition, we performed Pearson correlation and simple regression analysis to look for the relationships between corneal spherical aberration and asphericity. *P* values less than 0.05 were considered statistically significant.

## 3. Results

One hundred and thirty-four patients participated in this study, with 70 subjects that had SMILE and the other 64 subjects that had FS-LASIK. Detailed baseline characteristics by group are given in Table 1. There were no significant differences in any of the variables between the SMILE and FS-LASIK groups (*P* > 0.05).

### 3.1. Visual Acuity and Refraction

At 6 months postoperatively, the UDVA and CDVA were −0.12 ± 0.11 and **−**0.05 ± 0.05 in the SMILE group and −0.10 ± 0.06 and −0.03 ± 0.06 in the FS-LASIK group, respectively. The postoperative SE was **−**0.16 ± 0.19 D and −0.08 ± 0.25 D after SMILE and FS-LASIK, respectively. There was no difference between the two groups in the postoperative UDVA (*P* = 0.21), CDVA (*P* = 0.12), or SE (*P* = 0.08).

### 3.2. Corneal Spherical Aberration

**Figure 1:**
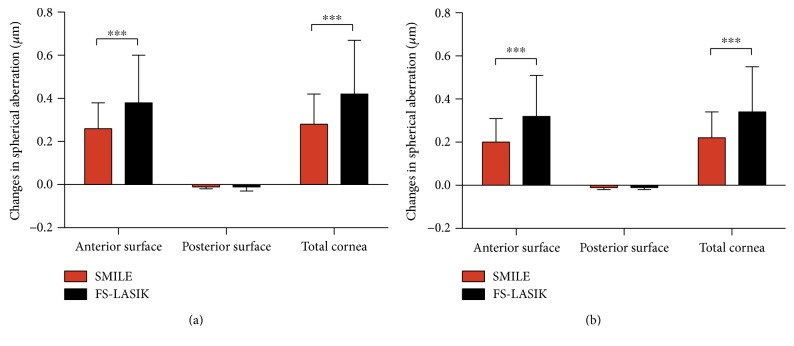
Changes in spherical aberrations of the anterior surface, the posterior surface, and the total cornea following SMILE and FS-LASIK at 1 (a) and 6 (b) months. SMILE showed less increases in the spherical aberration of the anterior surface and the total cornea than FS-LASIK at both 1 and 6 months. While no significant difference between SMILE and FS-LASIK in the changes of the spherical aberration of the posterior corneal surface was found. ∗∗∗ indicates significant differences between SMILE and FS-LASIK with *P* < 0.001.

For spherical aberrations of the anterior surface and the total cornea, a similar variation trend was found after SMILE and FS-LASIK, with an increase at 1 month, after which a decrease by 6 months (all *P* < 0.001). However, the magnitude of increases in the spherical aberration of the anterior surface and the total cornea was smaller after SMILE than after FS-LASIK at both 1 and 6 months (*P* < 0.001). With regard to spherical aberration of the posterior corneal surface, a little bit of increase was shown in both groups (*P* < 0.001). There was no significant difference between SMILE and FS-LASIK in the amount of changes in spherical aberration of the posterior corneal surface postoperatively (*P* > 0.05) (Table 2, Figure 1).

### 3.3. Corneal Asphericity

**Figure 2:**
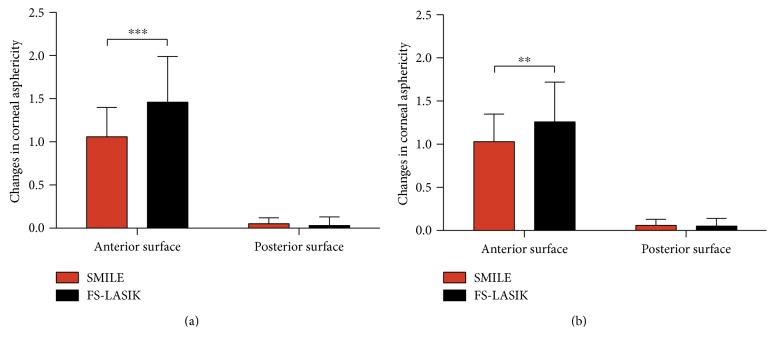
Changes in corneal asphericity of the anterior and posterior corneal surfaces after SMILE and FS-LASIK at 1 (a) and 6 (b) months. The increase in the *Q* value of anterior corneal surface was smaller after SMILE than after FS-LASIK. There was no significant difference between two groups in the changes of *Q* value of posterior corneal surface. ∗∗ indicates significant differences between groups with *P* < 0.01. ∗∗∗ indicates significant differences between groups with *P* < 0.001.

The *Q* value before surgery was negative for both the anterior and the posterior corneal surfaces. After SMILE and FS-LASIK, the *Q* value of the anterior corneal surface significantly shifted to positive value (SMILE: 0.84 ± 0.34 at 1 month, 0.81 ± 0.32 at 6 months; FS-LASIK: 1.21 ± 0.53 at 1 month, 1.01 ± 0.46 at 6 months) (all *P* < 0.001). The *Q* value of the posterior corneal surface significantly increased from preoperative −0.12 ± 0.11 to −0.07 ± 0.12 at 1 month and −0.06 ± 0.11 at 6 months after SMILE (all *P* < 0.001) and from preoperative −0.11 ± 0.13 to −0.07 ± 0.12 at 1 month and −0.06 ± 0.12 at 6 months after FS-LASIK (*P* = 0.03 and 0.001, resp.). Comparison between groups indicated SMILE had less increase in the *Q* value of anterior corneal surface than FS-LASIK (*P* < 0.001 at 1 month and *P* = 0.001 at 6 months, resp.). No significant difference in the change of *Q* value of posterior corneal surface was found between SMILE and FS-LASIK (*P* = 0.21 at 1 month and *P* = 0.26 at 6 months, resp.) (Figure 2).

### 3.4. Correlation and Regression Analysis

**Figure 3:**
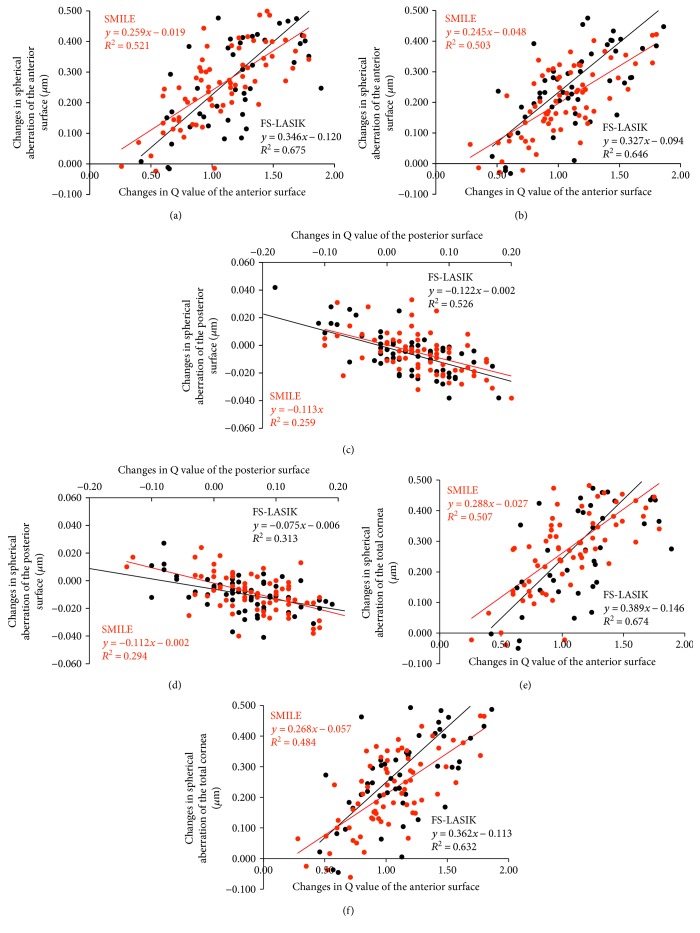
Changes in corneal spherical aberrations plotted as a function of changes in corneal asphericity. Significant linear correlation and regression were found at both 1 (a, c, e) and 6 months (b, d, f) after SMILE and FS-LASIK.

Data of correlation analysis by group at 1 and 6 months postoperatively are shown in Figure 3. After both the SMILE and FS-LASIK procedures, the changes in spherical aberrations of the anterior surface and the total cornea were positively correlated with the increase in *Q* value at the anterior corneal surface (all *P* < 0.001), and the changes in spherical aberrations of the posterior surface were negatively correlated with the increase in *Q* value at the posterior corneal surface (all *P* < 0.001). Simple linear regression models were conducted, with equations being shown in Figure 3.

## 4. Discussion

The present study investigated the visual acuity, refraction, and corneal spherical aberration following SMILE and FS-LASIK procedures. Our results showed that eyes achieved promising and similar visual and refractive outcomes in terms of UDVA, CDVA, and SE with treatment of SMILE and FS-LASIK. This indicated that both of these two procedures were effective in managing myopia and myopic astigmatism, which was consistent with previous reports [12]. Nonetheless, we found that the amount of changes in spherical aberration was smaller after SMILE than after FS-LASIK for the anterior surface and the total cornea, but not for the posterior corneal surface. Ocular spherical aberration changes have been compared between SMILE and FS-LASIK in previous studies. Lin et al. reported that SMILE induced fewer spherical aberrations when compared with FS-LASIK at 1 and 3 months of follow-up [13]. Liu et al. reported that 6 months postoperatively, SMILE had significantly lower induction of spherical aberrations (0.12 ± 0.22 *μ*m) than FS-LASIK (0.28 ± 0.26 *μ*m) for a 6.0 mm zone [14]. With regard to corneal spherical aberrations, Gyldenkerne et al. investigated the corneal spherical aberration changes over a 5.0 mm zone and found FS-LASIK induced 0.13 *μ*m and 0.14 *μ*m higher spherical aberrations of the anterior corneal surface and the total cornea as compared to SMILE [4]. Wu and Wang demonstrated that the changes of the anterior surface and the total cornea were significantly lower after SMILE surgery than after FS-LASIK surgery 3 months postoperatively [5]. More recently, Ye et al. compared the changes of anterior corneal higher order aberrations between SMILE and FS-LASIK 6 months after surgery and reported that SMILE induced significantly fewer total higher order aberrations and spherical aberration compared with FS-LASIK [7]. Our data were in accordance with these studies and indicated that SMILE might provide better visual quality than FS-LASIK.

In addition, we evaluated corneal shape changes described as corneal asphericity after myopic correction by SMILE and FS-LASIK. We found that the anterior corneal surface shifted from a prolate shape to an oblate shape following both SMILE and FS-LASIK and SMILE tended to induce less *Q* value increases than FS-LASIK along the anterior corneal surface at 1 and 6 months. This was consistent with study by Su et al., although only changes at 1 week postoperatively were compared in their study [15]. Gyldenkerne et al. demonstrated recently that the anterior corneal curvature after SMILE is steeper in the central 2 mm, but flatter in the periphery, than that after FS-LASIK, suggesting that SMILE better preserved the corneal asphericity [4]. In this study, we provided direct evidence for their speculation.

We considered possible explanations for differences in anterior corneal asphericity changes between SMILE and FS-LASIK. First, it is likely that the profile of the lenticule created in SMILE differs from the tissue ablation made in FS-LASIK. There might be potential different algorithms used during SMILE procedure. Second, for an excimer laser, the ablation efficiency reduction in the periphery of cornea would increase corneal asphericity even when the exact Munnerlyn ablation profile was used [16]. With respect to SMILE, the lenticule was created with femtosecond laser scanning at two depths of the stroma, which may avoid the ablation efficiency reduction in the periphery and therefore induce less increase in corneal asphericity. Third, it has been suggested that corneal epithelial remodeling plays a role in shape modulation after refractive surgery and that an increase in central epithelial thickness results in a shift toward increased oblateness [17, 18]. Recently, a comparative study reported that epithelial thickness increased less in the central zone and more in the midperiphery after SMILE than after FS-LASIK [19]. This may partly explain the observation of less asphericity increase of the anterior corneal surface after SMILE in this study.

For the posterior corneal surface, although a mild increase in *Q* value was observed for both the SMILE and FS-LASIK groups, the postoperative *Q* value was still negative, indicating that the posterior corneal surface preserved its prolate shape after SMILE and FS-LASIK. We speculated that the biomechanical changes to the cornea might be the plausible reason for the asphericity changes at the posterior corneal surface. As proposed by Roberts and Dupps [20, 21], central ablation can reduce the tension in residual peripheral lamellar segments. As a result, an outward peripheral force pulls laterally on the center, resulting in central corneal flattening after myopic ablation. Our study showed that SMILE induced similar posterior corneal shape changes as FS-LASIK surgery.

Another finding of our study was that the changes in spherical aberrations were dramatically correlated with the increase in *Q* value for both SMILE and FS-LASIK procedures. Especially for the anterior corneal surface, increase in *Q* value could lead to 50.3% and 64.6% of the variation in corneal spherical aberration following SMILE and FS-LASIK, respectively, even at a 6-month follow-up. This may provide potential insights into how to best optimize visual quality in SMILE by modifying the corneal asphericity. However, values of *R*^2^ revealed in regression models were not high enough to completely predict the changes, suggesting that there were other possible factors affecting the corneal spherical aberration. For example, corneal biomechanical properties [5] and treatment decentration [22] had been reported to be associated with the induced spherical aberration.

In summary, both SMILE and FS-LASIK exhibited excellent visual results and refractive outcomes. SMILE induced less increases in spherical aberration of the anterior corneal surface and the total cornea and better preserved the corneal asphericity of the anterior corneal surface than FS-LASIK. The changes of corneal asphericity could partly account for the variation of corneal spherical aberration after these two procedures.

## Figures and Tables

**Table 1:** Preoperative characteristics by group.

Parameters	SMILE (*n* = 70)	FS-LASIK (*n* = 64)	*P* value
Age (years)	22.16 ± 3.87	23.22 ± 3.62	0.10
Gender (n)			0.64
Male	40	34	
Female	30	30	
UDVA (logMAR)	1.09 ± 0.30	1.17 ± 0.30	0.15
CDVA (logMAR)	0.00 ± 0.00	0.00 ± 0.01	0.32
Sphere (D)	−5.52 ± 1.21	−5.86 ± 1.46	0.15
Cylinder (D)	−0.62 ± 0.48	−0.68 ± 0.42	0.46
SE (D)	−5.83 ± 1.23	−6.20 ± 1.52	0.13
Corneal spherical aberration (*μ*m)			
Anterior surface	0.25 ± 0.07	0.23 ± 0.07	0.23
Posterior surface	−0.15 ± 0.02	−0.16 ± 0.02	0.07
Total cornea	0.21 ± 0.08	0.18 ± 0.07	0.08
Corneal asphericity			
Anterior surface	−0.22 ± 0.09	−0.25 ± 0.10	0.07
Posterior surface	−0.12 ± 0.11	−0.11 ± 0.13	0.49

SMILE: small incision lenticule extraction; FS-LASIK: femtosecond laser-assisted laser in situ keratomileusis; UDVA: uncorrected distance visual acuity; CDVA: corrected distance visual acuity; D: diopters; SE: spherical equivalent.

**Table 2:** Spherical aberrations of the anterior surface, the posterior surface, and the total cornea in SMILE and FS-LASIK (*μ*m).

Groups	Preoperative	1 month	6 months	*P* value
SMILE				
Anterior surface	0.25 ± 0.07	0.51 ± 0.13^∗^	0.45 ± 0.11^∗^	<0.001
Posterior surface	−0.15 ± 0.02	−0.16 ± 0.03^†^	−0.16 ± 0.03^∗^	<0.001
Total cornea	0.21 ± 0.08	0.48 ± 0.15^∗^	0.42 ± 0.12^∗^	<0.001
FS-LASIK				
Anterior surface	0.23 ± 0.07	0.62 ± 0.22^∗^	0.55 ± 0.19^∗^	<0.001
Posterior surface	−0.16 ± 0.02	−0.16 ± 0.03^†^	−0.17 ± 0.02^∗^	<0.001
Total cornea	0.18 ± 0.07	0.60 ± 0.25^∗^	0.53 ± 0.21^∗^	<0.001

SMILE: small incision lenticule extraction; FS-LASIK: femtosecond laser-assisted laser in situ keratomileusis. ^∗^Significantly different from preoperative data at *P* < 0.001. ^†^Significantly different from preoperative data at *P* < 0.05.
